# Role of sympathetic pathway in light-phase time-restricted feeding-induced blood pressure circadian rhythm alteration

**DOI:** 10.3389/fnut.2022.969345

**Published:** 2022-09-08

**Authors:** Tianfei Hou, Aaron N. Chacon, Wen Su, Yuriko Katsumata, Zhenheng Guo, Ming C. Gong

**Affiliations:** ^1^Department of Physiology, College of Medicine, University of Kentucky, Lexington, KY, United States; ^2^Department of Pharmacology and Nutritional Sciences, College of Medicine, University of Kentucky, Lexington, KY, United States; ^3^Department of Biostatistics, College of Public Health, University of Kentucky, Lexington, KY, United States; ^4^Research and Development, Lexington Veterans Affairs Medical Center, Lexington, KY, United States

**Keywords:** blood pressure circadian rhythm, time-restricted feeding, sympathetic nervous system, heart rate variability, baroreflex, norepinephrine, alpha-adrenergic

## Abstract

Disruption of blood pressure (BP) circadian rhythm, independent of hypertension, is emerging as an index for future target organ damage and is associated with a higher risk of cardiovascular events. Previous studies showed that changing food availability time alters BP rhythm in several mammalian species. However, the underlying mechanisms remain largely unknown. To address this, the current study specifically investigates (1) the relationship between rhythms of food intake and BP in wild-type mice; (2) effects of light-phase time-restricted feeding (TRF, food only available during light-phase) on BP circadian rhythm in wild-type and diabetic *db/db* mice; (3) the roles of the autonomic system and clock gene in light-phase TRF induced changes in BP circadian rhythm. Food intake and BP of C57BL/6J and *db/db* mice were simultaneously and continuously recorded using BioDAQ and telemetry systems under *ad libitum* or light-phase TRF. Per2 protein daily oscillation was recorded *in vivo* by IVIS spectrum in mPer2*^Luc^* mice. Autonomic nerve activity was evaluated by heart rate variability, baroreflex, urinary norepinephrine (NE) and epinephrine (Epi) excretion, and mRNA expressions of catecholamines biosynthetic and catabolic enzymes, and alpha-adrenergic receptors in mesenteric resistance arteries. We found that in wild-type mice, the BP level was correlated with the food intake temporally across the 24 h. Reversing the feeding time by imposing light-phase TRF resulted in reverse or inverted BP dipping. Interestingly, the net changes in food intake were correlated with the net alteration in BP temporally under light-phase TRF. In *db/db* mice, light-phase TRF worsened the existing non-dipping BP. The food intake and BP circadian rhythm changes were associated with alterations in Per2 protein daily oscillation and the time-of-day variations in heart rate variability, baroreflex, and urinary excretion of NE and Epi, and increased mRNA expression of *Slc6a2* (encoding NE transporter) and *Adra1d* (encoding alpha-adrenergic receptor 1d) in the mesenteric resistance arteries, indicating the sympathetic nervous system (SNS) was modulated after light-phase TRF. Collectively, our results demonstrated that light-phase TRF results in reverse dipping of BP in wild-type and diabetic *db/db* mice and revealed the potential role of the sympathetic pathway in light-phase TRF-induced BP circadian rhythm alteration.

## Introduction

Normal blood pressure (BP) exhibits a circadian rhythm that rises in the morning and decreases 10–20% during the night, known as dipping BP. Disruptions of BP circadian rhythm, which often manifest as non-dipping BP (less than 10% decrease of nighttime BP from daytime BP) or reverse or inverted dipping BP (higher nighttime than daytime BP), are highly prevalent in patients with hypertension, type 2 diabetes, chronic kidney disease, and sleep apnea syndrome (1, 2). Clinical studies demonstrate that non-dipping and reverse dipping BP is associated with target organ damages and increased detrimental cardiovascular events (1, 3, 4). Thus, understanding the mechanisms that control normal BP circadian rhythm has high clinical relevance.

Experimentally limiting food availability only to the inactive phase alters BP rhythm markedly in several mammalian species (5–8), suggesting that the timing of food intake plays a critical role in regulating BP circadian rhythm. However, these studies did not simultaneously monitor the food intake and BP circadian rhythm. Hence, whether or not food intake temporally correlates with BP circadian rhythm and directly triggers BP change under altered feeding schedule remains unclear. Nocturnal animals consume most food during the dark phase when they are active under normal conditions. The current study alters the feeding schedule to the light phase, the inactive phase of nocturnal mice, termed as light-phase time-restricted feeding (light-phase TRF) and aims to address these important issues and investigate whether food intake temporarily correlates with BP alteration by simultaneously monitoring the episodic feeding activity by the BioDAQ system and the beat-to-beat BP by telemetry in singly housed mice under *ad libitum* feeding (ALF) or light-phase TRF.

Among the multiple mechanisms regulating BP homeostasis, the critical role of the sympathetic nervous system (SNS) is well recognized. SNS activity, measured by plasma or urine catecholamine levels, exhibits circadian rhythms that parallel BP rhythm (9, 10). Previous studies demonstrated that SNS activity is suppressed during fasting and enhanced after feeding (11–15). Consistent with these studies, we recently reported that the non-dipping BP is associated with diminished rhythms of food intake and SNS activity in diabetic *db/db* mice (16). Importantly, we demonstrated that aligning food availability with the standard light-dark cycle by dark-phase TRF, which limits food availability only to the active dark phase, restores BP and SNS activity rhythms in *db/db* mice (16). However, whether SNS activity rhythm serves as the mechanistic linkage between light-phase TRF and BP circadian rhythm alteration has not been investigated. The current study tested if the modulation of SNS activity mediates, at least in part, the light-phase TRF-induced BP circadian rhythm alterations in wild-type mice. In addition, we recently reported that dark-phase TRF protects BP circadian rhythm in diabetic *db/db* mice (16). However, the effect of light-phase TRF on BP circadian rhythm in diabetic *db/db* mice remains to be illustrated. The current study also investigated whether, in contrast to the dark-phase TRF-induced protection of BP circadian rhythm (16), light-phase TRF worsens the non-dipping BP in diabetic *db/db* mice.

The molecular basis of circadian rhythm is the endogenous autonomous clocks present in the central suprachiasmatic nucleus and nearly all peripheral tissues. The intrinsic clocks, comprised of a group of transcription factors that form a feedback loop, are entrained by the environment and thus maintained in a ∼24-h rhythm (17). While light is the most potent environmental cue to entrain the central clock, accumulating evidence indicates that food intake is a major factor in entraining peripheral clocks (17). Mouse models harboring clock gene mutation or knockout suggest that clocks play a critical role in regulating BP circadian rhythms (18). To investigate the potential involvement of clocks in light-phase TRF-induced alteration of BP circadian rhythm, we, therefore, examined the oscillation of Period 2 (per2), one of the core clock genes, in response to light-phase TRF using luciferase knock-in mice (Per2*^luc^*) (19).

Collectively, the current study aims to elucidate (1) the temporal relationships between food intake and BP circadian rhythm in wild-type mice fed *ad libitum* and light-phase TRF; (2) the responses of the SNS and Per2 oscillation to light-phase TRF in wild-type mice; (3) the effects of light-phase TRF on BP circadian rhythm in *db/db* mice.

## Materials and methods

### Animals

C57BL/6J (Stock No.: 000664), homozygous mPer2*^Luc^* (Stock No: 006852), and *db/db* (Stock No.: 000642) mice were purchased from the Jackson Laboratory (Bar Harbor, ME, United States). Only male mice were used in the current study. The mice were housed under a 12:12 light: dark cycle in a light-tight box and fed with a standard rodent diet with free water access. All animal procedures were approved by the Institutional Animal Care and Use Committee of the University of Kentucky.

### Time-restricted feeding and food intake monitoring

Food intake of 17-week-old wild-type mice (*n* = 7) or 21-week-old *db/db* mice (*n* = 10) were recorded under ALF (*ad libitum*) for 3 days, followed by 7 days’ of light-phase TRF (10 h of food access from zeitgeber time (ZT) 2 to ZT12). The time of food availability and the amount of food consumed were recorded by a BioDAQ system (Research Diet, New Brunswick, NJ) (16). The BioDAQ system has a feeding module fitted with an electronic sensor and an automated gate controller, allowing food access at a designated time and continuous accurate monitoring of food intake. Mice were acclimated to the system for at least 7 days before the experiment, and food intake at different time intervals was analyzed as indicated in the figures.

### Measurement of blood pressure, heart rate, and locomotor activity with telemetry

The same wild-type and *db/db* mice used in the food intake monitoring experiment were implanted with a telemetry probe (TA11PA-C10, Data Sciences International, St. Paul, MN, United States) into the left common carotid artery to continuously record BP, heart rate, and locomotor activity in free-moving mice at a sampling rate of 1,000 Hz (16, 20, 21). Mice were allowed 7–10 days of recovery from surgery before measurement.

### *In vivo* imaging of mPer2 time-of-day variation in the kidney and liver

Seventeen-week-old mPer2*^luc^* mice (*n* = 17) under ALF were imaged at zeitgeber time ZT5, ZT11, ZT17, and ZT23 and then subjected to light-phase TRF and imaged again on day 3 and day 7 using the IVIS system (IVIS Spectrum *in vivo* imaging system, PerkinElmer, Waltham, MA, United States) as previously described (22). Briefly, mice were anesthetized with 2.5–4% isoflurane and subcutaneously injected with D-luciferin (15 mg/kg body weight in PBS). The mice were imaged 7 min later for dorsal side up and 10 min later for ventral side up for 5 s using the IVIS spectrum. Total bioluminescence (photon/s/cm2/sr) was quantified by setting the region of interest to the same shape and size using Living Image software (IVIS Imaging System). To eliminate individual mouse and measurement variation and to better quantify the phase of Per2 oscillation, relative bioluminescence intensity was calculated by normalizing absolute bioluminescence to the average bioluminescence of the four-time points (22).

### Heart rate variability analysis

Heart rate variability was analyzed using frequency and time domain methods by Ponemah Software (Data Sciences International; St. Paul, MN) as previously described (16). Briefly, for frequency domain determination, 2-min artifacts-free beat-by-beat BP waveform segments were selected from every 20 min across the 72 h of recordings for the final analysis. Each segment was then interpolated to 20Hz using the quadratic method, followed by Fast Fourier Transformation using hanning window method. The cut-off frequency ranges for low-frequency (LF) and high-frequency (HF) were 0.15–0.6 Hz and 1.5–4 Hz, respectively. For time-domain analysis, 5-min beat-by-beat BP waveform segments over 72 h were calculated, and the root-mean-square successive beat-to-beat difference (rMSSD) was plotted as the parasympathetic heart rate control marker. Systolic pressure was used as the trigger for frequency domain and time domain analysis. The heart rate variability was averaged in each correspondent hour over 3 days for both the frequency and time domain data to generate a 24-h heart rate variability profile.

### Cardiac baroreflex sensitivity analysis

Cardiac baroreflex sensitivity (referred to as baroreflex sensitivity in the manuscript for simplicity) was analyzed using sequence techniques by Hemolab software^1^ as previously described (16, 21). For each hour, the software searches sequences in which the systolic arterial pressure and pulse interval were positively correlated (*r*^2^ > 0.80) to identify valid sequences with at least four consecutive changes as an effective Baroreflex. The average slope of the systolic pressure-pulse interval relationships is calculated as baroreflex sensitivity. For each mouse, 72 hourly baroreflex sensitivity data points were calculated from the three consecutive days of BP data and then averaged to generate one 24-h profile.

### Urine collection and catecholamines measurement

Urine was collected using metabolic cages (Tecniplast, West Chester, PA). To prevent mice from dehydration and urine contamination by food crumbs, the mice were fed a gel diet (DietGel76A, ClearH_2_O, Portland, ME) during the urine collection period. The mice were acclimated to the metabolic cage and gel diet for 3 days before actual urine collection. The 12-h light- and dark-phase urine samples were first collected in 15- to 16-week-old mPer2*^Luc^* mice (*n* = 11) under ALF. Then the mice were subjected to light-phase TRF for 7 days, and the 12-h light- and dark-phase urine samples were collected again on the last day of light-phase TRF. We used mPer2*^Luc^* mice because mPer2*^Luc^* mice have been backcrossed to C57BL/6J inbred mice for 11 generations, and mPer2*^Luc^* mice exhibited a similar normal food intake and BP rhythm as C57BL/6J mice (22). Urinary norepinephrine (NE) and epinephrine (Epi) were determined by the ELISA kits (Abnova, Taipei, Taiwan). Total contents of NE and Epi were calculated by concentrations × urine volumes (16).

### Quantitative analysis of mRNA expression

Twenty-week-old male Per2*^Luc^* mice were randomly divided into ALF (*n* = 5) or light-phase TRF (*n* = 6) groups.7 days later, mice were euthanized between ZT9 and ZT11, and mesenteric arteries were collected in RNAlater solution (ThermoFisher Scientific, United States). The fat surrounding the mesenteric arteries was carefully removed under a dissecting microscope. As previously described (16, 21, 23–25), the mRNA levels of various genes were quantified by real-time PCR. The real-time PCR primers for each gene were described in Supplementary Table 1.

### Statistical analysis

The sample size was determined based on previous publications (16, 26). All data were expressed as mean ± standard error of the mean (SEM). For comparison of 1 parameter in the same mice, a paired t-test was performed. For analysis of 1 parameter in different mice, an unpaired t-test was used. Repeated two-way ANOVA with matching conditions between light vs. dark phase and between ALF vs. light-phase TRF with Tukey’s post-test was conducted to compare two parameters in the same mice. The main factors for two-way ANOVA were feeding (ALF vs. light-phase TRF) and time (light phase vs. dark phase or feeding vs. fasting). Correlations between food intake and BP and between Δfood intake/Δlocomotor activity and ΔBP were calculated using linear regression. An ANCOVA was performed where feeding method (ALF vs. light-phase TRF) and light condition (light vs. dark) were used as factors, and BP and locomotor activity under ALF or light-phase TRF were used as dependent variable and covariant respectively. All statistical analyses, expect ANCOVA, were performed by Prism 9 software (GraphPad Software, San Diego, CA). ANCOVA was performed by IBM SPSS Statistics. *P* < 0.05 was defined as statistically significant.

## Results

### Food intake rhythm correlates with blood pressure rhythm in mice under *ad libitum* feeding

To determine the temporal relationship between food intake and BP under ALF and light-phase TRF, 16-week-old male wild-type C57BL/6J mice were implanted with telemetry and acclimated in BioDAQ cages under ALF for 10 days, followed by light-phase TRF for 7 days. During light-phase TRF, food was available from ZT2 to ZT12 (ZT0: lights-on and ZT12: lights-off). Food intake and BP were monitored simultaneously and continuously in the same single-housed mouse using BioDAQ and telemetry systems during the last 3 days of ALF and 7 days of light-phase TRF.

**FIGURE 1:**
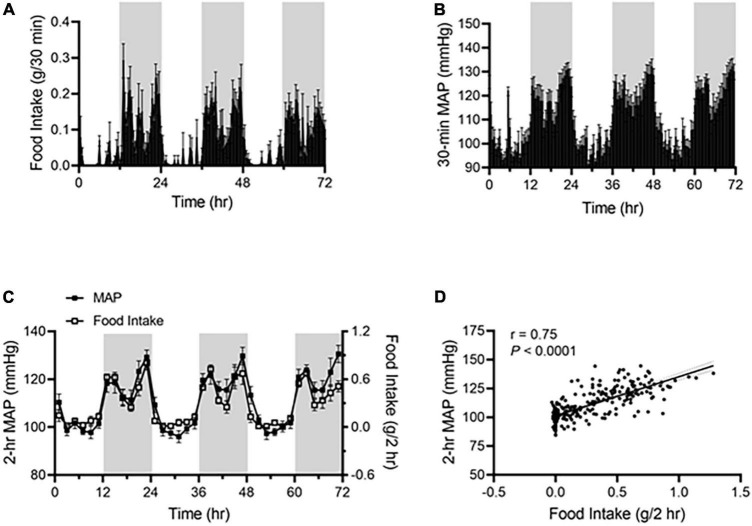
Food intake correlates with BP rhythm in *ad libitum-fed* mice. Food intake and BP were recorded by BioDAQ and telemetry in 17-week-old male C57BL/6J mice (*N* = 7). **(A,B)** Daily profiles of accumulated food intake **(A)** and average mean arterial pressure [MAP; **(B)**] in 30-min (min) intervals over 72-h during the light and dark phases, shown in white and gray, respectively. **(C)** Average MAP (left axis) and accumulated food intake (right axis) in 2-h intervals over 72-h during the light and dark phases. **(D)** Linear regression analysis of average MAP and accumulated food intake in 2-h intervals. Data were expressed as the mean ± standard error of the mean (SEM).

Under ALF, wild-type mice consumed more food during the dark phase than during the light phase (Figure 1A), and their mean arterial pressure (MAP) was higher during the dark phase than during the light phase (Figure 1B). The two parameters showed a highly similar pattern when 2 h-average MAP was plotted with 2 h-sum food intake during the corresponding time on the same graph (Figure 1C). There was a significant correlation between food intake and MAP, with a linear correlation coefficient of *r* = 0.75 (Figure 1D).

### Light-phase time-restricted feeding rapidly alters blood pressure rhythm and results in reverse dipping

**FIGURE 2:**
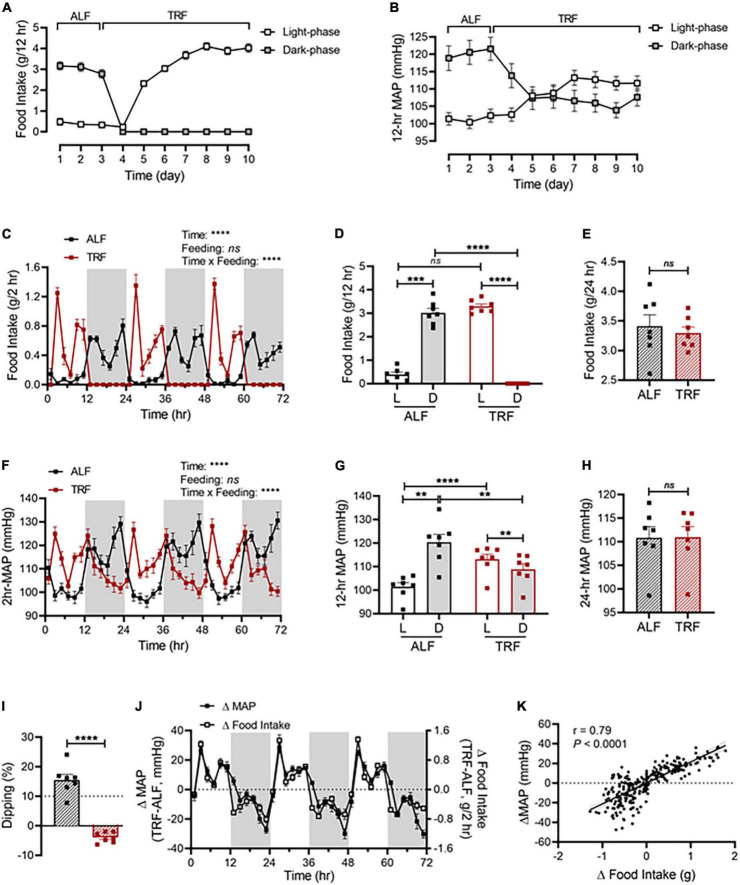
Light-phase TRF rapidly alters BP rhythm. **(A,B)** Accumulated food intake **(A)** and average MAP **(B)** during the light and dark phase in 17-week-old male C57BL/6J mice (*N* = 7) under 3 days of ALF, followed by 7 days of TRF. **(C–E)** Accumulated food intake in 2-h **(C)**, 12-h **(D)**, and 24-h **(E)** intervals over 3 days under ALF and during the last 3 days under light-phase TRF. **(F–H)** Average MAP in 2-h **(F)**, 12-h **(G)**, and 24-h **(H)** intervals over 3 days of ALF and the last 3 days under light-phase TRF. **(I)** MAP dipping with ALF and reverse dipping with light-phase TRF. **(J)** Net changes in accumulated food intake (Δfood intake) and average MAP (ΔMAP) in 2-h intervals over 72 h. **(K)** Linear regression of Δfood intake and ΔMAP. Data were expressed as the mean ± SEM and analyzed by repeated two-way ANOVA with matching conditions between light vs. dark phase and between ALF vs. light-phase TRF with Tucky’s *post-hoc* analysis **(C,D,F,G)**, paired *t*-test **(E,H,I)**, and simple linear regression **(K)**.**P < 0.01; ***P < 0.001; ****P < 0.0001; ns, not significant.

As shown in Figure 2A, light-phase TRF started with food withdrawal on day 4 during the dark phase, leading to the shift of food consumption mostly during the dark phase (day 1-3) to exclusively during the light phase (day 4-10). In parallel with the shift in food intake, a rapid decrease in the dark-phase MAP occurred during the first day of light-phase TRF (day 4; Figure 2B) and nearly reached a steady state by the second day of light-phase TRF (day 5; Figure 2B). In contrast, an increase in the light-phase food intake and MAP was observed on the second day of light-phase TRF and reached a steady state on the fourth day of light-phase TRF (day 7; Figures 2A,B).

Light-phase TRF induced a phase shift of food intake and MAP from the dark phase to the light phase (Figures 2C,F). Cosinor analysis showed that the acrophase of MAP was shifted from ZT19.2 ± 0.2 with ALF to ZT9.7 ± 0.3 after light-phase TRF (Supplementary Table 2). Consequently, there was a significant increase in the food intake and MAP during the light phase and a significant decrease in the food intake and MAP during the dark phase, respectively (Figures 2D,G). As a result, the light-dark phase MAP difference under ALF was reversed under light-phase TRF (Figure 2G) and the amplitude of MAP oscillation was decreased after light-phase TRF compared to ALF (Supplementary Table 2). When analyzing the dipping status, light-phase TRF resulted in reverse-dipping of BP (Figure 2I). In contrast, light-phase TRF did not affect 24-h average MAP (Figure 2H). Interestingly, the 24-h daily food intake was comparable between ALF and light-phase TRF (Figure 2E).

To further define whether changes in MAP during light-phase TRF temporally correlate with the changes in food intake, we calculated the net differences in food intake and MAP between ALF and light-phase TRF by subtracting food intake or MAP during ALF from light-phase TRF. We found that the net differences in food intake were almost completely parallel to the net difference in MAP (Figure 2J), indicating a change in food intake can timely trigger a change in MAP. Linear regression analysis revealed a significant correlation between Δfood intake and ΔMAP (Figure 2K). Light-phase TRF also induced similar changes in SBP (Supplementary Figures 1A–D) and DBP (Supplementary Figures 1E–H).

Telemetry also recorded heart rate and locomotor activity in mice under ALF or light-phase TRF. Similar to its effects on BP, light-phase TRF also induced similar changes in locomotor activity (Supplementary Figures 2A-C) and heart rate (Supplementary Figures 2D-F). Notebly, the net changes in locomotor activity also parallel to the net differences in MAP (Supplementary Figure 2G) and linear regression analysis showed a significant correlation between Δlocomotor activity and ΔMAP (*r* = 0.79, Supplementary Figure 2H). To further evaluate whether alterations in locomoter activity mediates light-phase TRF induced BP changes, we analyzed the effects of light-phase TRF on MAP after adjusting for locomotor activity using ANCOVA. The result showed that locomotor activity is a covariate [F (1,23) = 6.618, p = 0.017], however, the feeding method (ALF vs. TRF) still has a significant effect on BP even after adjusted for locomotor activity [F (1,23) = 4.64, p = 0.042].

### Light-phase time-restricted feeding advances Per2 protein daily oscillations

**FIGURE 3:**
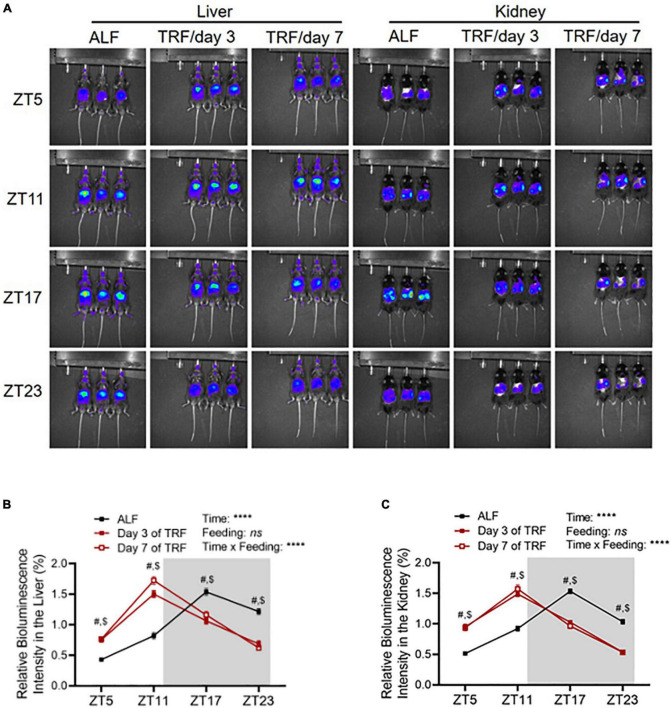
Light-phase TRF shifts Per2 protein daily oscillation. **(A)** Representative images of Per2 bioluminescence in the liver and kidney in 17-week-old male mPer2*^Luc^* mice under ALF and at day 3 and day 7 after light-phase TRF. **(B,C)** Relative Per2 bioluminescence intensity in the liver **(B)** and kidney **(C)** under ALF and day 3 and day 7 after light-phase TRF. Data were expressed as the mean ± SEM (*N* = 17) and analyzed by repeated two-way ANOVA with matching conditions between ZT and between ALF vs. light-phase TRF with Tucky’s *post–hoc* analysis **(B,C)**. #, ALF *vs.* day 3 after TRF, *P* < 0.0001. $, ALF *vs.* day 7 after TRF, *P* < 0.0001.

To explore whether clocks are involved in light-phase TRF-induced BP circadian rhythm alteration, we determined Per2 protein expression by *in vivo* imaging in the liver and kidney in 17-week-old male mPer2*^Luc^* mice at ZT5, ZT11, ZT17, and ZT23 under ALF and on day 3 and day 7 of light-phase TRF using IVIS Spectrum. Under ALF, Per2 bioluminescence peaked at ZT17 during the dark phase in the liver and kidney (Figures 3A-C). Light-phase TRF for 3 days advanced Per2 bioluminescence for 6 h in the liver and kidney, with the highest at ZT11 during the light phase (Figures 3A-C). Prolonged light-phase TRF from 3 to 7 days did not further shift the Per2 bioluminescence phase in the liver and kidney since no difference was observed in Per2 protein diurnal variations between day 3 and day 7 under light-phase TRF (Figures 3A-C).

### Light-phase time-restricted feeding alters the sympathetic pathway

**FIGURE 4:**
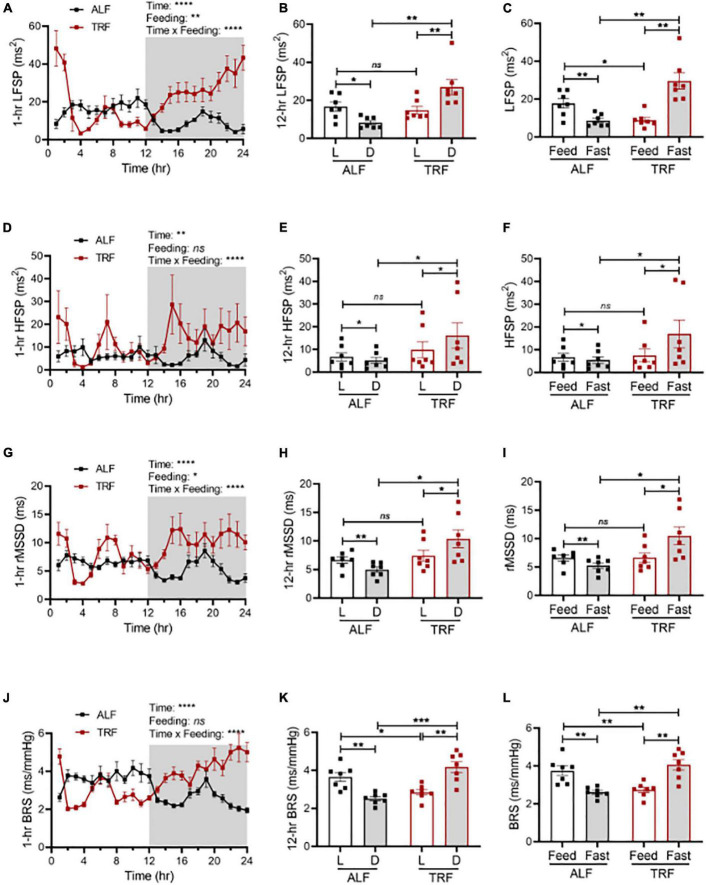
Light-phase TRF modulates autonomic nervous activity. Heart rate variability and cardiac baroreflex sensitivity (BRS) were calculated from BP data recorded 3 days under ALF and the last 3 days of TRF. *N* = 7. **(A,B)** Low-frequency spectral power (LFSP) in 1-h **(A)** and 12-h **(B)** intervals. **(C)** LFSP during feeding and fasting period. **(D,E)** High-frequency spectral power (HFSP) in 1-h **(D)** and 12-h **(E)** intervals. **(F)** HFSP during feeding and fasting period. **(G,H)** Root mean square of successive differences between normal heartbeats (rMSSD) in 1-h **(G)** and 12-h **(H)** intervals. **(I)** The rMSSD during the feeding and fasting period. **(J,K)** BRS in 1-h **(J)** and 12-h **(K)** intervals. **(L)** BRS during feeding and fasting period. Data were expressed as the mean ± SEM and analyzed by repeated two-way ANOVA with matching conditions between light vs. dark phase or feed vs. fast and between ALF vs. light-phase TRF with Tucky’s *post–hoc* analysis **(A–L)**. *P < 0.05; **P < 0.01; ***P < 0.001; ****P < 0.0001; ns, not significant.

To explore whether light-phase TRF might modify the sympathetic nervous system (SNS), thus altering BP circadian rhythm in wild-type mice, we first calculated the heart rate variability (HRV) in the same groups of ALF- and light-phase TRF-fed 17-week-old wild-type C57BL/6J mice described above. HRV was calculated by frequency and time domain measurements as the low-frequency spectral power (LFSP), high-frequency spectral power (HFSP), and root means square of successive RR interval differences (rMSSD). Under ALF, LFSP was lower in the dark phase than in the light phase (Figures 4A,B). Light-phase TRF did not change the average LFSP during the 12-h light phase but significantly increased LFSP during the 12-h dark phase (Figure 4B). Because food was available only for 10 h during the 12-h light phase under light-phase TRF, we also analyzed the HRV during the fasting (ZT2 to ZT12) *vs.* feeding (ZT13 to ZT2) period in mice under ALF and light-phase TRF to depict food intake-induced LFSP changes accurately. Interestingly, light-phase TRF *vs.* ALF significantly decreased the LFSP during the feeding period but increased the LFSP during the fasting period (Figure 4C). A similar result was also obtained from the HRV analysis of HFSP and rMSSD during the dark/fasting phase but not during the light/feeding period (Figures 4D-I).

We next determined spontaneous cardiac baroreflex sensitivity (BRS) in the same groups of ALF- and light-phase TRF-fed 17-week-old wild-type C57BL/6J mice described above. Results showed that mice have higher BRS during the light phase than during the dark phase under ALF (Figures 4J,K). Light-phase TRF significantly decreased BRS during the light phase (Figure 4K) or the feeding period (Figure 4L) but increased BRS during the dark phase (Figure 4K) or the fasting period (Figure 4L).

**FIGURE 5:**
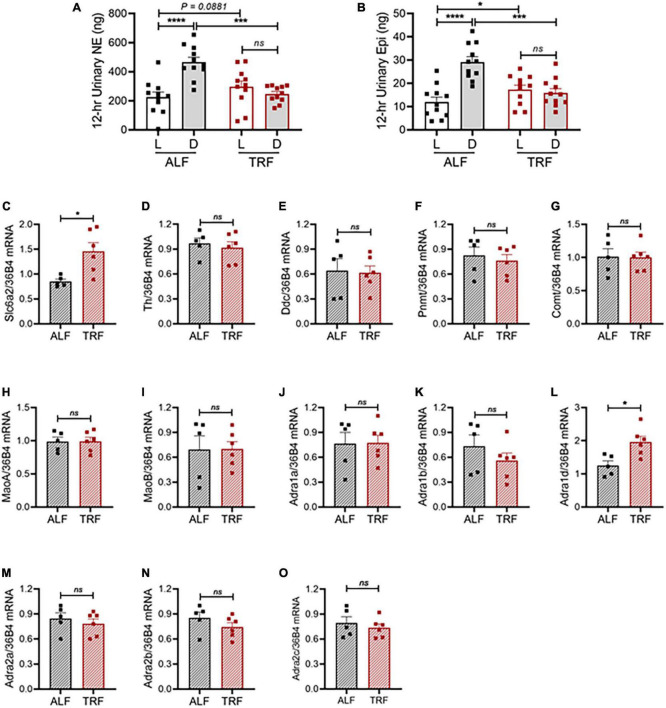
Light-phase TRF alters the sympathetic pathway. **(A,B)** Urinary norepinephrine [NE; **(A)**] and epinephrine [Epi; **(B)**] during the light and dark phases in 15- to 16-week-old male mPer2*^Luc^* mice (*N* = 11) under ALF and the 7th day of light-phase TRF. **(C–O)** mRNA expression of solute carrier family 6 member 2 [(*Slc6a2*, **(C)**], tyrosine hydroxylase [*Th*, **(D)**], dopa decarboxylase [*Ddc*, **(E)**], phenylethanolamine *N*-methyltransferase [*Pnmt*, **(F)**], catechol-O-methyltransferase [*Comt*, **(G)]**, monoamine oxidase A [*MaoA*, **(H)**], monoamine oxidase B [*MaoB*, **(I)**], alpha-1a adrenergic receptor [*Adra1a*, **(J)**], alpha-1b adrenergic receptor [*Adra1b*, **(K)**], alpha-1d adrenergic receptor [*Adra1d*, **(L)**], alpha-2a adrenergic receptor [*Adra2a*, **(M)**], alpha-2b adrenergic receptor [*Adra2b*, **(N)**], and alpha-2c adrenergic receptor [*Adra2c*, **(O)**] in mesenteric arteries isolated during ZT9 to ZT11 from 20-week-old male mPer2*^Luc^* mice under ALF and 7 days after light-phase TRF. *N* = 5 or 6 in each group. Data were expressed as the mean ± SEM and analyzed by repeated two-way ANOVA with matching conditions between light vs. dark phase and between ALF vs. light-phase TRF with Tucky’s *post hoc* analysis **(A,B)** or unpaired t-test **(C–O)**. *P < 0.05; ***P < 0.001; ****P < 0.0001; ns, not significant.

To more directly evaluate the role of the SNS in light-phase TRF-induced BP circadian rhythm alteration, we analyzed urinary norepinephrine (NE) and epinephrine (Epi) excretion during the light and dark phases in 15- to 16-week-old male mPer2*^Luc^* mice under ALF and light-phase TRF. The 12-h light- and dark-phase urine samples were collected in metabolic cages on day 4 of ALF and day 7 of light-phase TRF. Results showed that urinary NE and Epi exhibited a diurnal variation that is significantly lower during the light phase than during the dark phase in mice under ALF (Figures 5A,B), which coincided with BP rhythm (i.e., Figure 2G). Also, in line with its effect on BP circadian rhythm alteration, light-phase TRF significantly suppressed urinary NE and Epi during the dark phase (Figures 5A,B). In addition, light-phase TRF also had a trend of increased urinary NE and a significant increased urinary Epi excretion during the light phase (Figures 5A,B). As a result, diurnal variations of urinary NE and Epi excretion were abolished in mice under light-phase TRF (Figures 5A,B).

To investigate the molecular mechanism by which light-phase TRF induces BP circadian rhythm alteration, we determined mRNA expressions of the genes responsible for catecholamine biosynthesis, catabolism, and function in mesenteric resistant arteries in 20-week-old male mPer2*^Luc^* mice under ALF and 7 days after light-phase TRF. To rule out the potential effect of the timing on the gene expression, mice were euthanized at the same time (ZT9-ZT11). Interestingly, light-phase TRF significantly increased the expression of *carrier family 6 member 2 (Slc6a2)*, a NE transporter (NET) gene, but did not affect expressions of *tyrosine hydroxylase (Th)* and *dopa decarboxylase (Ddc)*, two NE biosynthesis enzyme genes, and *phenylethanolamine N-methyltransferase* (*Pnmt*), *catechol-O-methyltransferase* (*Comt*), and *monoamine oxidase A and B (MaoA and MaoB)*, three NE catabolism genes (Figures 5C–I). Also of interest is the light-phase TRF upregulated alpha *1d adrenergic receptor* (*Adra1d) gene* but not other subtype adrenergic receptors, including alpha *1a adrenergic receptor* (*Adra1a)*, alpha *1b adrenergic receptor* (*Adra1b)*, alpha *2a adrenergic receptor* (*Adra2a)*, alpha *2b adrenergic receptor* (*Adra2b)*, and alpha *2c adrenergic receptor* (*Adra2c)* (Figures 5J–O).

### Light-phase time-restricted feeding worsens blood pressure rhythm and blood glucose in diabetic *db/db* mice

Given that light-phase TRF reverses BP circadian rhythm in non-diabetic C57BL/6J mice (Figure 2), we hypothesized that light-phaseTRF was detrimental rather than beneficial to BP circadian rhythm in *db/db* mice. To test this hypothesis, 21-week-old wild-type male *db/db* mice were implanted with telemetry and acclimated in BioDAQ cages under ALF for 10 days, followed by light-phase TRF for 7 days. Food intake and BP were monitored simultaneously and continuously during the last 3 days of ALF and 7 days of light-phase TRF.

**FIGURE 6:**
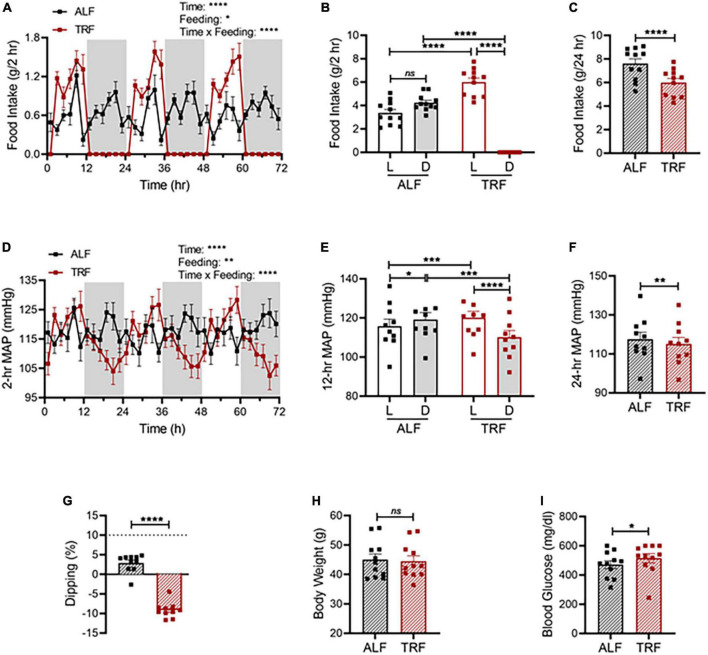
Light-phase TRF worsens BP rhythm and blood glucose in *db/db* mice. **(A–C)** Accumulated food intake in 2-h **(A)**, 12-h **(B)**, and 24-h **(C)** intervals in 21-week-old male *db/db* mice (*N* = 10) over 3 days of ALF and during the last 3 days of TRF. **(D–F)** Average MAP in 2-h **(D)**, 12-h **(E)**, and 24-h **(F)** intervals over 3 days with ALF or during the last 3 days of TRF. **(G)** Non-dipping MAP (less than 10%) with ALF and reverse dipping with light-phase TRF. **(H,I)** Body weight **(H)** and blood glucose **(I)** were determined at ZT1 under ALF and after 7 days of TRF. Data were expressed as the mean ± SEM and analyzed by repeated two-way ANOVA with matching conditions between light vs. dark phase and between ALF vs. light-phase TRF with Tucky’s *post hoc* analysis **(A,B,D,E)** or paired *t*-test **(C,F,G–I)**. *P < 0.05; **P < 0.01; ****P < 0.0001; ns, not significant.

Compared with non-diabetic C57BL/6J mice, diabetic *db/db* mice under ALF exhibit dampened circadian rhythms in food intake (Figures 6A vs. 2C) and MAP (Figures 6D vs. 2F), lost the diurnal difference in food intake (Figures 6B vs. 2D) and MAP (Figures 6E vs. 2G), and were non-dippers (Figures 6G vs. 2I). Importantly, light-phase TRF significantly increased the food intake (Figures 6A,B) and MAP (Figures 6D,E) during the light phase while decreasing them during the dark phase in *db/db* mice (Figures 6A,B,D,E), resulting in reverse dipping BP (Figure 6G). Similar to its effect on MAP, light-phase TRF also induced a similar detrimental impact on SBP (Supplementary Figures 3A-D), DBP (Supplementary Figures 3E-H), locomotor activity (Supplementary Figures 4A-C), and heart rate (Supplementary Figures 4D-F). In contrast to its little effect on 24-h total food intake and average MAP in non-diabetic C57BL/6J mice (Figures 2E,H), light-phase TRF significantly reduced 24-h total food intake and average MAP in diabetic *db/db* mice (Figures 6C,F). Interestingly, despite its reduced food intake and daily MAP, light-phase TRF did not affect the body weight of *db/db* mice (Figure 6H), but significantly elevated blood glucose level in diabetic *db/db* mice (Figure 6I).

## Discussion

The current study demonstrated that (1) the BP level correlated with the food intake temporally across the 24 h in C57BL/6J mice under ALF; (2) light-phase TRF rapidly reverted BP circadian rhythm, leading to reverse dipping; (3) light-phase TRF-induced net changes in food intake temporally correlated with the net changes in BP; (4) light-phase TRF-induced BP rhythm alteration was associated with alterations in the time-of-day variations in Per2 protein expression, heart rate variability, baroreflex sensitivity, and urinary excretion of NE and Epi; (5) light-phase TRF increased mRNA expression of *Slc6a2* and *Adra1d* during the light phase in mesenteric arteries; and (6) light-phase TRF worsened BP dipping and increased blood glucose in diabetic *db/db* mice.

In contrast to the extensive studies linking TRF to metabolic health, there are limited studies that examined the effect of TRF on BP circadian rhythm. A few publications on this topic suggest the timing of food intake is critical for BP circadian rhythm. In nocturnal rats and mice, a higher BP during the dark phase than during the light phase parallels higher food consumption during the dark phase. Experimentally restricting food availability to a period during the normal fasting/inactive phase is associated with an increase in BP during the fasting/inactive phase in rabbits (5), dogs (6), rats (7), and mice (8). However, these studies measured the total food intake during the entire feeding period or the 12-h light and dark phase. Thus, whether food intake temporally correlates with BP change is unclear. In the current study, we simultaneously monitored food intake every 1-min using BioDAQ and BP continuously using telemetry in a single housed mouse. These state-of-art technologies allow us to define the temporal relationship between food intake and BP in a single-housed mouse. Our results demonstrated for the first time that the BP level correlated with the food intake temporally across the 24 h in C57BL/6J mice under ALF (Figure 1).

We recently reported restricting food availability to 8 or 12 h during the dark/active phase (dark-phase TRF) protected the BP circadian rhythm in diabetic *db/db* mice (16). However, whether the light-phase TRF has similar protection for diabetic *db/db* mice has not been investigated. In contrast to dark-phase TRF (16), the current study illustrated that light-phaseTRF rapidly exacerbated rather than protected *db/db* mice from disruption of BP circadian rhythm (Figure 6 and Supplementary Figures 3, 4). Also, in contrast to the dark-phase TRF that did not influence BP circadian rhythm in non-diabetic wild-type mice (16), the current study demonstrated that light-phase TRF rapidly reversed BP circadian rhythm in C57BL/6J mice, leading to reverse or inverted dipping (Figure 2 and Supplementary Figure 1). Moreover, the current study also showed light-phase TRF-induced net changes in food intake are temporally correlated with net changes in BP in C57BL/6J mice (Figure 2K), indicating a causal role of food intake in regulating BP circadian rhythm in mice. In line with the previous reports (5–8, 16), these results suggest that TRF could be beneficial or detrimental to BP circadian rhythm depending upon the timing of food intake.

The potent influence of the timing of food intake on BP circadian rhythm could have significant relevance to human health. In modern society, more and more people consume food during the entire 16 h of awake time, including night hours (27). This erratic eating rhythm is associated with increased metabolic syndrome/diabetes and non-dipping or reversed dipping BP (28). In these patients, the reverse dipping BP is often caused by an increase in dark-phase/nighttime BP (29–39). However, some patients have a decline in active-phase/daytime BP in addition to a rise in inactive-phase/nighttime BP (40–46). The reverse dipping BP caused by light-phase TRF in wild-type mice described in the current study seems to resemble more of the latter group of patients. Further investigation is warranted to verify this. In addition, postprandial hypotension, a common cause of falls, syncope, and stroke is common among the elderly, reaching a prevalence of 25–67% in institutionalized elders (47). Impaired regulation of eating associated BP changes likely contribute to the cause. It will be interesting to test whether old mice would develop postprandial hypotension and serve as a model to dissect the underlying mechanism.

How does TRF trigger alterations in BP circadian rhythm? While complex mechanisms are likely underlying light-phase TRF-induced alteration in BP circadian rhythm, the current study provides several lines of evidence for a potentially important role of the SNS. The results are in line with our previous study showed that dark-phase TRF protects BP circadian rhythm via suppressing sympathetic nervous activity during fasting in diabetic *db/db* mice (16).

Firstly, our power spectral analysis of HRV, an index of the autonomic activity (48, 49), demonstrated that light-phase TRF increased LFSP, HFSP, and rMSSD during the dark/fasting phase but decreased LFSP or did not affect HFSP and rMSSD during the light/feeding period (Figures 4A-I). It is generally accepted that HFSP and rMSSD reflect parasympathetic activity (50). In contrast, the interpretation of LFSP is inconsistent. Some considered it a sympathetic modulation marker, whereas others considered it a parameter of sympathetic and parasympathetic activity (51). There were also studies claiming that during resting conditions, LFSP primarily reflects baroreflex activity but not sympathetic innervation (50, 52, 53). Regardless of these different interpretations, the current study is consistent with our recent report (16), light- or dark-phase TRF modulates BP circadian rhythm, at least in part, through the auto autonomic nervous system, including the SNS.

Secondly, the baroreceptor reflex system plays a predominant role in preventing BP fluctuations by modulating both SNS and PNS activities and therefore reflects the overall integrity of the autonomic nervous system (54). Under physiological conditions, baroreceptors are constantly active and continuously inhibit SNS activity. Under pathological conditions, such as hypertension, coronary artery disease, myocardial infarction, and heart failure, baroreflex control is impaired, with an imbalance of sympathetic−vagal outflow (55). Our results that BRS decreased during the light/feeding phase but increased during the dark/fasting phase after light-phase TRF (Figures 4J-L) indicates feeding, in addition to light, modulate sympathetic-vagal outflow thus can contribute to the changes in BP rhythm.

Thirdly, the current study showed that urinary NE and Epi were increased during the light phase but decreased during the dark phase after light-phase TRF, abolishing the day-to-night NE and Epi variations (Figures 5A,B). These results are commensurate with our recent report in *db/db* mice (16) and other studies that SNS activity, measured by sympathetic firing rate in brown adipose tissue in rats fed a high-fat diet (11), NE turnover in the heart in normal rats (12), renal sympathetic nerve activity in cats (14), urinary catecholamines and their metabolites excretion in humans (13), indicating that eating or feeding enhances SNS activity whereas fasting reduces it.

Fourthly, the current study revealed that light-phase TRF increased the mRNA expression of *Slc6a2* without affecting NE synthesis or catabolic enzymes in mesenteric resistant arteries (Figures 5C-I). *Slc6a2* encodes a NET protein responsible for removing NE from synapses by uptaking NE back into the presynaptic nerve terminal, the rate-limiting step to terminate NE function (56). It has been shown that increasing catecholamines by chronic depolarization (57) or stimulating TH activity (58) are associated with NET mRNA upregulation. Thus, enhanced mRNA expression of NET after light-phase TRF suggests that catecholamine synthesis/release may be increased. Nevertheless, the lack of change in NE metabolic enzymes at the mRNA level does not exclude possible alterations in catecholamine synthesis because acute stimulation of NE synthesis is mostly achieved by regulating TH activity through post-translational phosphorylation (59). Further experiments are needed to test these possibilities.

Lastly, one of the intriguing findings from the current study is that light-phase TRF selectively increased the mRNA expression of *Adra1d* among six subtypes of αARs (α_1*A*_-, α_1*B*_-, α_1*D*_-, α_2*A*_-, α_2*B*_-, and α_2*C*_-AR) in mesenteric resistant arteries (Figures 5J-O). *Adra1d* encodes an αAR protein responsible for regulating physiopathological responses mediated by NE and Epi, particularly in cardiovascular diseases, including hypertension (60). Mice deficient in *Adra1a* and *Adra1d* are hypotensive (61, 62), whereas mice deficient in *Adra1b* are normotensive (63). All three subtypes of α_1_ARs knockout mice have decreased vasopressor response (61–63), but only *Adra1a* or *Adra1d* knockout mice have reduced aortic vascular contractility to NE and Epi (61, 62). Consistent with these genetic studies, pharmacological studies showed that α_1*D*_-AR selective antagonist BMY7378 inhibits phenylephrine-induced vasoconstriction in rat aorta (64–66) and decreases BP (64). Therefore, it is tempting to speculate that the finding that increased *Adra1d* mRNA expression in mesenteric resistant arteries in response to light-phase TRF may indicate that light-phase TRF alters BP circadian rhythm, at least in part, through *Adra1d*. Further experiments are also needed to explore this possibility.

In addition to the SNS, other mechanisms may also participate in light-phase TRF-induced BP circadian rhythm alteration. Locomotor activity is a well-known factor for BP regulation. Our data showed that light-phase TRF promoted significant alterations in locomotor activity, which is highly correlated with the net changes in BP and locomotor activity acts as a covariant in light-phase TRF-induced BP alteration. Clocks, including period circadian regulator 2 (Per2), are well recognized for regulating BP homeostasis and circadian rhythms (16, 20, 67–69). To determine the timeline changes of Per2 expression, *in vivo* Per2*^Luc^* bioluminescence was determined in mPer2*^Luc^* mice because mPer2*^Luc^* mice have a luciferase reporter gene fused to the endogenous mPer2 gene, thus allowing *in vivo* monitoring of the Per2 clock protein oscillation in response to light-phase TRF (19). A previous study showed that mPer2*^Luc^* mice are viable and fertile, with no developmental or morphological differences compared to wild-type littermates (19). We found that light-phase TRF-induced BP circadian rhythm alteration was associated with Per2*^Luc^* bioluminescence in the liver and kidney (Figure 3). Consistent with this notion, it has been reported that mice with Per2 mutation exhibited impaired endothelium-dependent relaxations in the aorta and non-dipping BP in mice with stimulated renin-angiotensin signaling by Ang II infusion (70–72). However, it is worth mentioning that a recent study showed that light-phase TRF altered BP circadian rhythm in mice independent of Bmal1 (8), indicating that the clock gene is not involved. Thus, whether Per2 is involved in light-phase TRF-induced BP alteration remains elusive.

In conclusion, the current study demonstrated that light-phase TRF results in reverse dipping of BP in both wild-type and diabetic *db/db* mice, and the SNS pathway plays a potential role in mediating light-phase TRF-induced BP circadian rhythm alterations.

## Data availability statement

The original contributions presented in this study are included in the article/Supplementary material, further inquiries can be directed to the corresponding authors.

## Ethics statement

The animal study was reviewed and approved by Institutional Animal Care and Use Committee of the University of Kentucky.

## Author contributions

TH, AC, and WS performed the experiments. TH analyzed the data. YK evaluated statistical analysis of the data. TH, ZG, and MG contributed to the idea, experimental design, and writing and editing of the manuscript. AC contributed to the editing of the manuscript. All authors contributed to the article and approved the submitted version.

## Footnotes

1
http://www.haraldstauss.com/HemoLab/HemoLab.html
