# Supplementary material for: Role of sympathetic pathway in light-phase time-restricted feeding-induced blood pressure circadian rhythm alteration
Source: Front Nutr. 2022 Sep 8;9:969345. doi: 10.3389/fnut.2022.969345 (PMC9493072; doi:10.3389/fnut.2022.969345)
Supplement: Supplementary file 1 [file Data_Sheet_1.PDF]

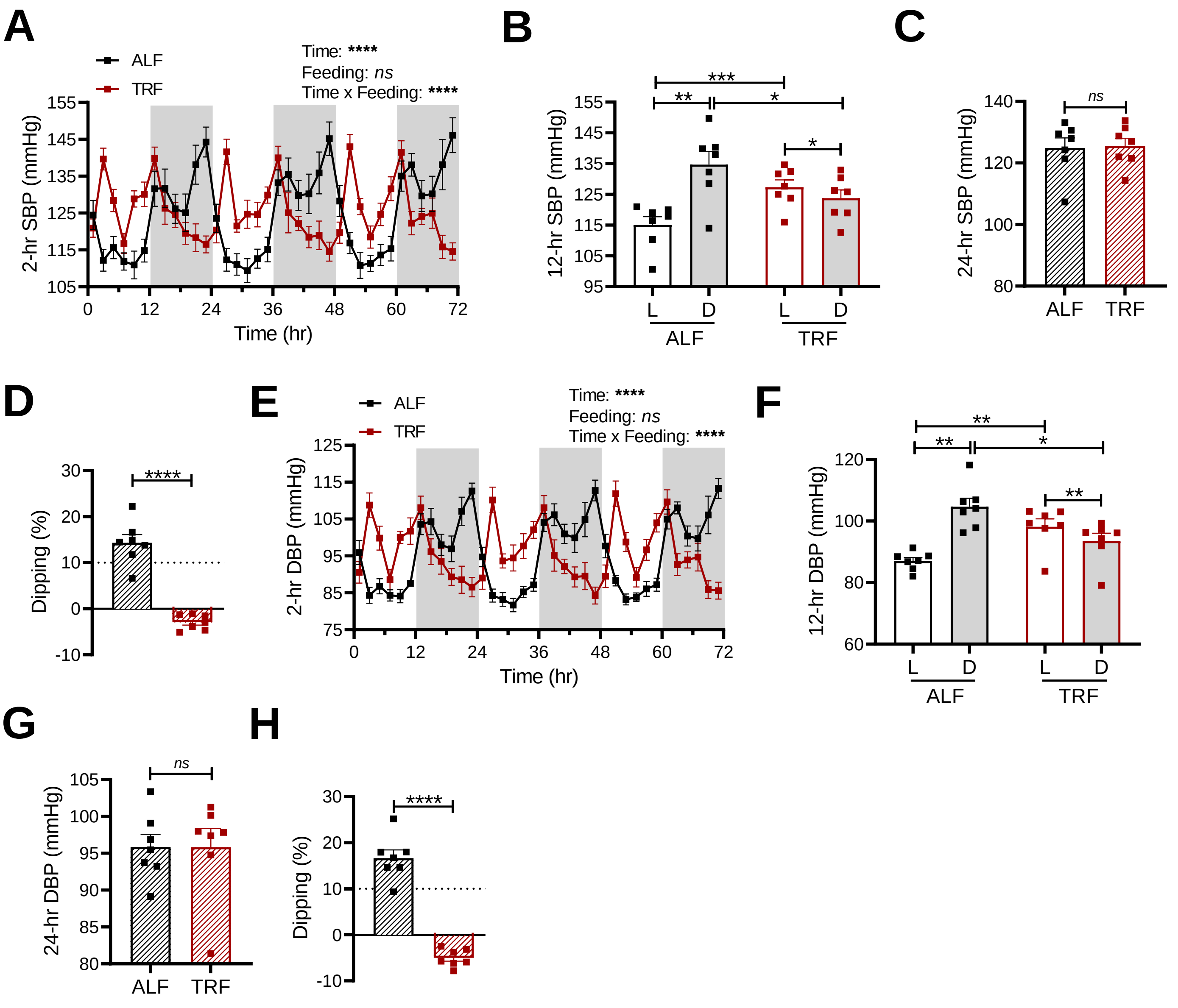

Supplemental Figure 1. Light-phase TRF alters SBP and DBP circadian rhythm. **(A−C)** Average systolic blood pressure (SBP) in 2-hr (A), 12-hr (B), and 24-hr (C) intervals in 17-week-old male C57BL/6J mice (N = 7) over 3 days of ALF and the last 3 days of light-phase TRF. **(D)** SBP dipping with ALF and reverse dipping with light-phase TRF. **(E−G)** Average diastolic blood pressure (DBP) in 2-hr (E), 12-hr (F), and 24-hr (G) intervals over 3 days of ALF and the last 3 days of light-phase TRF. **(H)** DBP dipping with ALF and reverse dipping with light-phase TRF. Data were expressed as the mean ± SEM and analyzed by repeated two-way ANOVA with matching conditions between light vs. dark phase and between ALF vs. light-phase TRF with Tucky's post hoc analysis (A, B, E, and F) and paired t-test (C, D, G, and H).*, *P* < 0.05; **, *P* < 0.01; ***, *P* < 0.001; ****, *P* < 0.0001; ns, not significant.

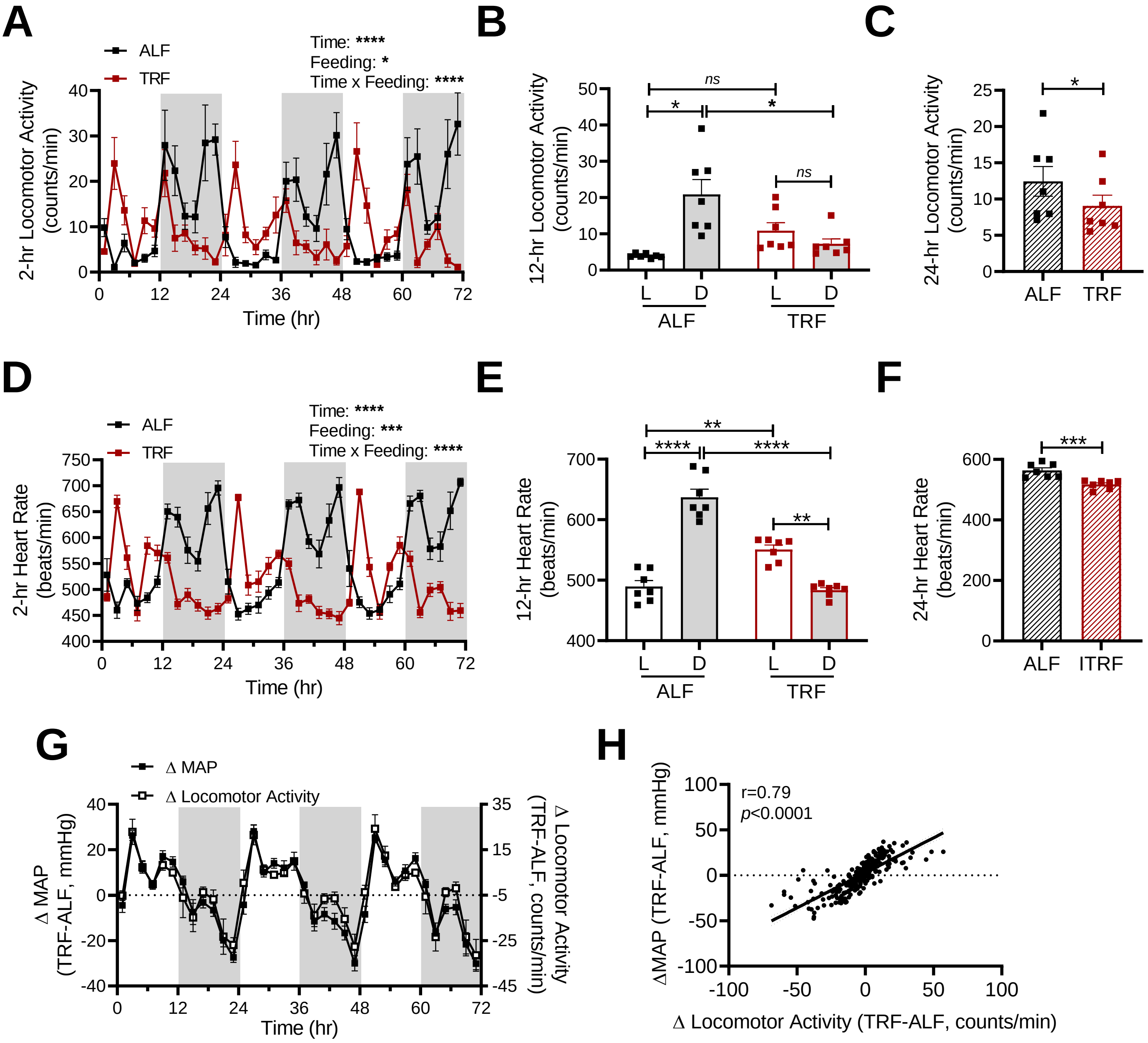

Supplemental Fig. 2 Light-phase TRF alters locomotor activity and heart rate rhythms. **(A−C)** Average locomotor activity in 2-hr (A), 12-hr (B), and 24-hr (C) intervals over 3 days in 17-week-old male C57BL/6J mice (N = 7) over 3 days of ALF and the last 3 days of light-phase TRF. **(D−E)** Average heart rate in 2-hr (D), 12-hr (E), and 24-hr (F) intervals over 3 days of ALF and the last 3 days of light-phase TRF. **(G)** Net changes in average locomotor activity (Δlocomotor activity) and MAP (ΔMAP) in 2-hr intervals over 72 hrs. **(H)** Linear regression of Δlocomotor activity and ΔMAP. Data were expressed as the mean ± SEM and analyzed by repeated two-way ANOVA with matching conditions between light vs. dark phase and between ALF vs. light-phase TRF with Tucky's post hoc analysis (A, B, D, and E) and paired t-test (C and F). *, $P < 0.05$; **, $P < 0.01$; ***, $P < 0.001$; ****, $P < 0.0001$; ns, not significant.

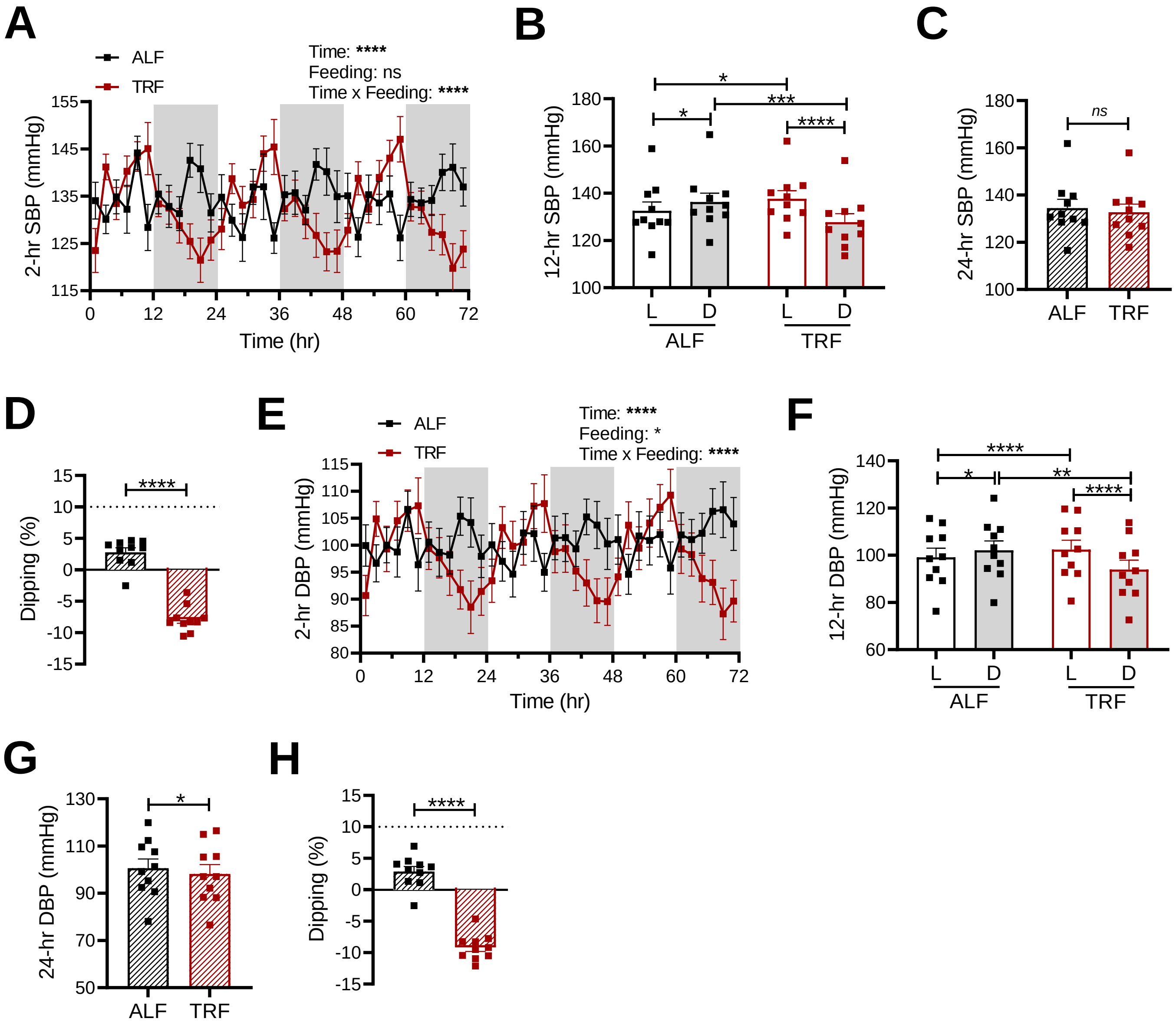

Supplemental Figure 3. Light-phase TRF worsens SBP and DBP circadian rhythm in *db/db* mice. **(A−C)** Average systolic blood pressure (SBP) in 2-hr (A), 12-hr (B), and 24-hr (C) intervals over 3 days intervals in 21-week-old male *db/db* mice (N = 10) over 3 days of ALF and the last 3 days of light-phase TRF. **(D)** Nondipping SBP (less than 10%) with ALF and reverse dipping with light-phase TRF. **(E−G)** Average diastolic blood pressure (DBP) in 2-hr (E), 12-hr (F), and 24-hr (G) intervals over 3 days of ALF and the last 3 days of light-phase TRF. **(H)** Nondipping DBP (less than 10%) with ALF and reverse dipping with light-phase TRF. Data were expressed as the mean ± SEM and analyzed by repeated two-way ANOVA with matching conditions between light vs. dark phase and between ALF vs. light-phase TRF with Tucky's post hoc analysis (A, B, E, and F) and paired t-test (C, D, G, and H). *, $P < 0.05$; **, $P < 0.01$; ***, $P < 0.001$; ****, $P < 0.0001$; ns, not significant.

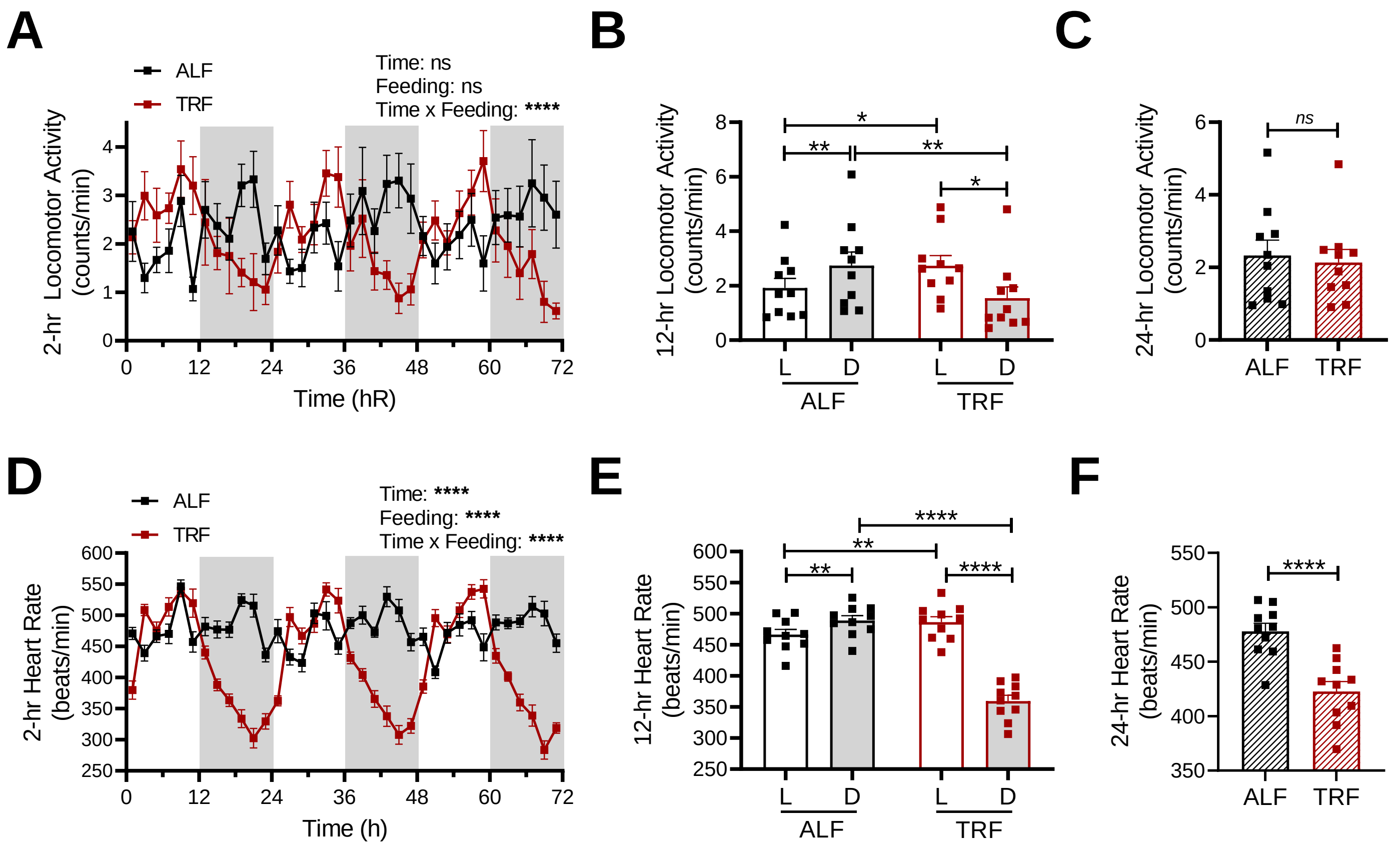

Supplemental Fig. 4 Light-phase TRF alters locomotor activity and heart rate rhythms. **(A−C)** Average locomotor activity in 2-hr (A), 12-hr (B), and 24-hr (C) intervals in 21-week-old male *db/db* mice (N = 10) over 3 days of ALF and the last 3 days of light-phase TRF. **(D−E)** Average heart rate in 2-hr (D), 12-hr (E), and 24-hr (F) intervals over 3 days of ALF and the last 3 days under TRF. Data were expressed as the mean ± SEM and analyzed by repeated two-way ANOVA with matching conditions between light vs. dark phase and between ALF vs. light-phase TRF with Tucky's post hoc analysis (A, B, D, and E) and paired t-test (C and F). *, $P < 0.05$; **, $P < 0.01$; ***, $P < 0.001$; ****, $P < 0.0001$; ns, not significant.

Supplemental Table 1. Real-time PCR primer information*

Gene	Primer	Sequence
Ddc	Forward	AGCTGGTTGCTTACACATCTG
	Reverse	CGAAGGGACTGCTTTTAGCTT
Th	Forward	TCTCCTTGAGGGGTACAAAACC
	Reverse	ACCTCGAAGCGCACAAAGT
Pnmt	Forward	CGCTGCATGGCACAAGTCT
	Reverse	GCCGGAGCCAATATCAATGA
Comt	Forward	CTGCCATCACCCAGCAAAT
	Reverse	CCCCGATGAGGATGGAAAC
MaoA	Forward	GGTCCTCCTTGGGGATAAAG
	Reverse	TCTCAGGTGGAAGCTCTGGT
MaoB	Forward	ATGAGCAACAAAAGCGATGTGA
	Reverse	TCCTAATTGTGTAAGTCCTGCCT
Slc6a2	Forward	TGTGGCGGTTCCCTTATCTCT
	Reverse	CCCCTCCCGGTTGTATTGC
Adra1a	Forward	CTGGGTCTTGGTCTTTGGAG
	Reverse	ATTTGCTGAGACCGAAGTGG
Adra1b	Forward	TCTTCATCGCTCTCCCACTT
	Reverse	GAACCCATGGCTTGACTCCA
Adra1d	Forward	TCCGTAAGGCTGCTCAAGTT
	Reverse	AGCGGGTTCACACAGCTATT
Adra2a	Forward	GGTGACACTGACGCTGGTTT
	Reverse	ACTGGTGAACACCGCGATAATA
Adra2b	Forward	TCTTCACCATTTTCGGCAATGC
	Reverse	AGAGTAGCCACTAGGATGTCG
Adra2c	Forward	CTGTGGTGGGTTTCCTCATCG
	Reverse	CCCGAAGTACCAGTAGGCCA

* All PCR primers are mouse-specific.

Supplemental Table 2. Cosinor analysis

	Parameter	ALF	Inactive-phase TRF	P value
MAP	Amplitude (mmHg)	12.6±1.7	5.8±0.7	0.0101*
	Acrophase (ZT)	19.2±0.2	9.7±0.3	<0.0001****
SBP	Amplitude (mmHg)	13.2±1.8	5.5±0.7	0.006**
	Acrophase (ZT)	19.3±0.2	9.9±0.3	<0.0001****
DBP	Amplitude (mmHg)	11.7±1.5	6.2±0.6	0.0227*
	Acrophase (ZT)	19.0±0.1	9.6±0.3	<0.0001****
LA[#]	Amplitude (mmHg)	10.0±2.1	3.9±1.2	0.0069**
	Acrophase (ZT)	19.2±0.3	8.9±1.1	<0.0001****
HR[#]	Amplitude (mmHg)	89.5±10.1	49.8±3.8	0.0099**
	Acrophase (ZT)	18.1±0.1	7.3±0.2	<0.0001****

[#] LA: Locomotor Activity; HR: Heart Rate.
